# Complications of radical hysterectomy with pelvic lymph node dissection for cervical cancer: a 10-year single-centre clinical observational study

**DOI:** 10.1186/s12885-022-10395-9

**Published:** 2022-12-08

**Authors:** Huang Jing, Wu Xiuhong, Yu Ying, Cheng Xiyun, Luo Deping, Shen Changmei, Wang Qi, Peng Tao, Pan Yiyun

**Affiliations:** 1 Department of Gynecology and Oncology, Ganzhou Cancer Hospital, Ganzhou, Jiangxi Province People’s Republic of China; 2 Radiotherapy Center, Ganzhou Cancer Hospital, Ganzhou, Jiangxi Province People’s Republic of China; 3 Chemotherapy Center, Ganzhou Cancer Hospital, No.19, HuaYuan Qian Road, Ganzhou, Jiangxi Province 341000 People’s Republic of China

**Keywords:** Cervical cancer, Radical hysterectomy, Pelvic lymph node dissection, Complications, Clinical observation

## Abstract

**Background and purpose:**

The complications of radical surgery for cervical cancer can increase patient suffering and affect their quality of life. This retrospective study assessed the safety of radical hysterectomy (RH) with pelvic lymph node dissection (PLND) by observing the complications of patients with cervical cancer who underwent this procedure in a single centre over 10 years. Our findings may provide experience and evidence for preventing and reducing complications.

**Methods:**

A total of 2226 cervical cancer patients who met the inclusion criteria were enrolled. All patients underwent RH + PLND. Intraoperative injury to adjacent tissues and short-term and long-term complications were recorded to analyze factors associated with the occurrence of complications.

**Results:**

Postoperative complications occurred in 34.41% (766/2226) of patients, including 7.68% of patients with injury to adjacent tissues, 31.45% with short-term complications, and 2.96% with long-term complications. Age, tumor size, invasion depth, parametrial invasion, lymph vascular space invasion (LVSI), lymph node metastasis, International Federation of Gynaecology and Obstetrics (FIGO) stage, and surgical procedure were closely associated with the postoperative complications of RH + PLND (*P* < 0.05).

**Conclusions:**

The results of this study showed that RH + PLND for cervical cancer is safe and practical. Patients aged 40–60 years, with tumors ≥ 4 cm, invasion depth ≥ 2/3, parametrial invasion, LVSI, lymph node metastasis, FIGO stage > IB2, and who underwent open surgery were more prone to complications.

## Introduction

Cervical cancer is the most common malignant tumour of the female reproductive system, with an incidence that has been increasing annually and a trend toward occurring in younger patients. This cancer currently ranks among the top gynaecological malignancies in developing countries [1, 2]. Multimodality therapy for cervical cancer includes surgery, radiotherapy, chemotherapy, and concurrent chemoradiotherapy [3, 4]. In addition, the research and application of neoadjuvant chemotherapy(NACT) have provided an increasing number of patients with cervical cancer the opportunity to undergo surgery [5, 6]. Although there are different treatment options for cervical cancer patients, under the premise of achieving therapeutic effects and in order to reduce treatment-related side effects, it is beneficial to avoid multi-modality treatment to reduce patients' pain and improve their quality of life.

Abdominal radical hysterectomy (RH) with pelvic lymph node dissection (PLND) is a classic surgical procedure used for the treatment of cervical cancer, which has demonstrated definite therapeutic efficacy [7, 8]. With the continuous advancement of surgical techniques and medical equipment, a growing number of radical surgeries for cervical cancer have progressed from open to minimally-invasive laparoscopic or robot-assisted laparoscopic surgery [9, 10]. Although the latter offers clear advantages, such as small surgical wounds and reduced pain, it also has disadvantages, including a limited operating space [11].Minimally invasive radical hysterectomy has a higher recurrence rate and a lower disease-free survival rate than open surgery, but the postoperative quality of life is similar, and open radical hysterectomy is recommended for patients with early-stage cervical cancer [12]. The Study Group for Gynecologic Oncology and the Study Group for Gynecologic Endoscopy of the German Society for Gynecology and Obstetrics disputed the results of the laparoscopic approach to cervical cancer(LACC) study, arguing that the inclusion criteria, short follow-up period, incomplete patient data, and learning curve influenced the results [13].The European Society of Gynecologic Oncology also considered the results of the LACC study to be unexpected, suggesting that tumor spread due to inadequate surgical scope and improper operation/CO2 ventilation may have contributed to poorer survival in the minimally invasive group [14]. J Minim and Lee also questioned the reliability of the LACC study results, suggesting that the design of the study in question was not rigorous enough and might lead to erroneous results [15, 16].

Numerous studies including preoperative neoadjuvant therapy, intraoperative radiotherapy, and postoperative adjuvant therapy have been conducted to reduce postoperative complications and improve survival in cervical cancer. Gupta [17] suggested that in stage IB2-IIB cervical cancer, NACT had a lower Disease-free survival (DFS) than concurrent radiotherapy group and no significant difference in overall survival (OS), but less toxic side effects. The application of NACT in cervical cancer remains controversial. NACT may not necessarily improve prognosis, but it can reduce the incidence of postoperative pathological risk factors and improve the chances of preserving fertility in young patients with early-stage squamous cervical cancer [18–20]. An meta-analysis reported that early response of NACT was associated with better DFS, and responders achieved a significantly higher survival rate than non-responders [21]. While the results of the two other Meta-analyses were dissimilar [4, 6].

Regardless of the surgical procedure, there is a risk of injury to adjacent organs and pelvic autonomic nerves during the operation, which can lead to postoperative complications and even permanent neurological sequelae or even lead to death, thus majorly impacting patient quality of life [22, 23].To reduce the incidence of complications during radical surgery for cervical cancer and improve the quality of life of cervical cancer patients, we retrospectively observed the complications of RH + PLND in a single centre over 10 years. Our findings may provide more clinical experience and evidence for reducing complications.

## Materials and methods

### Study design and study participants

This study design followed international regulations according to the Declaration of Helsinki. Our research was approved by the Ethical Committee of the Ganzhou Cancer Hospital (2,022,001) and written informed consent was obtained from participants.

This was a single center retrospective study, and all data were obtained from the case management center of Ganzhou Cancer Center. Patients with cervical cancer who met the inclusion criteria between January 2011 and December 2020 with complete clinical information and follow-up data were selected, and the information and data during hospitalization were recorded in the case, and the follow-up after discharge was done by the follow-up room of the case management center by telephone. Missed cases are not included in the enrollment criteria.

Patient inclusion criteria: (1) Patients aged 20–75 years; (2) no history of other malignant tumors; (3) newly diagnosed and treatment-naive patients with pathologically confirmed cervical cancer; (4) International Federation of Gynaecology and Obstetrics (FIGO) 2009 stages IA2–IIA2; (5) Karnofsky Performance Scale (KPS) score ≥ 80; (6) no serious organic diseases of the vital organs (e.g., the heart, liver, and lungs), and able to tolerate surgery; (7) patients who underwent RH + PLND; (8) surgeries performed by surgeons proficient in abdominal and laparoscopic RH + PLND; (9) with complete clinical data.

Patient exclusion criteria: (1) Non-epithelial cervical malignancy; (2) FIGO 2009 stages > IA2–IIA2; (3) patients with serious organic diseases of the vital organs (e.g., the heart, liver, and lungs), and unable to tolerate surgery; (4) patients with rectal and bladder dysfunction, such as difficulty in urination, urinary frequency, urinary urgency, urinary and fecal incontinence, and urinary tract infections; (5) history of abdominal and pelvic radiotherapy; (6) Patients with pre-operative NACT.

The criteria for minimally invasive surgery are: (1) IA2; (2) IB1, and tumor less than 2 cm; (3) patient financial support; (4) patient signed informed consent; (5) study center has complete equipment to perform minimally invasive surgery; (6) surgeon is skilled to perform open and laparoscopic extensive hysterectomy combined with pelvic lymph node dissection.

All cases in this study were operated on with RH + PLND. Ortholateral parametrial resection at the level of the medial iliac vessels, ventral parametrial resection at the level of the bladder, dorsal parametrial resection at the level of the rectum, and vaginal resection of 2 cm or as needed. Minimally invasive surgery was recommended for patients who met the criteria for minimally invasive surgery, when the patient's financial situation, the patient's informed consent and the center's equipment allow; and open surgery was performed for patients who met the criteria for minimally invasive surgery but did not agree to minimally invasive surgery and for patients who did not meet the criteria for minimally invasive surgery.

### Follow-up and collection of clinical data

A total of 2226 patients who met the inclusion criteria were enrolled; these patients were aged 20–72 years, with a median age of 51.83 years. Among them, 1254 patients underwent open surgery, 972 patients underwent laparoscopic surgery, 347 patients underwent postoperative radiotherapy, and 95 patients underwent postoperative concurrent chemoradiotherapy (Table 1).

**Table 1:** General clinical data of the patients included in this study (*n* = 2226)

Parameter	Number of patients, n (%)
Age, years	
20–40	298 (13)
41–50	710(32)
50–60	632 (29)
61–72	586 (26)
Other concomitant diseases	
Yes	637 (29)
No	1589 (71)
Pathological type	
Squamous cell carcinoma	2079 (93)
Adenocarcinoma	108(5)
Adenosquamous carcinoma	39 (2)
Pathological grade	
G1	135(6)
G2	1382(62)
G3	670(30)
Gx	39 (2)
Tumour size (cm)	
> 4	88(4)
≤ 4	2138(96)
FIGO stage	
IA2	310 (14)
IB1	1499 (67)
IB2	51 (2)
IIA1	329 (15)
IIA2	37 (2)
Surgical procedure	
Open surgery	1254 (56)
Laparoscopic surgery	972 (44)
Postoperative treatment	
No treatment	1784(80)
Radiotherapy	347(16)
Concurrent chemoradiotherapy	95(4)

The decision for ovary preservation was made for all patients according to their disease condition, age, and wishes. After RH with bilateral salpingo-oophorectomy, PLND was performed, which included the left and right common iliac, internal and external iliac, obturator, deep inguinal, and presacral lymph nodes. If necessary, the para-aortic lymph nodes were also excised for biopsy.

The patients’s mean follow-up time was 44.8 months (13.6–112.2 months). The perioperative observations mainly included ureteral injury, bladder injury, bowel injury, surgical wound dehiscence, surgical site infection, urinary tract infection, pelvic lymphocyte, and ureteral fistula. Long-term follow-up was conducted according to the principles of follow-up for malignant tumors. The observations included urinary dysfunction, bowel dysfunction, and pelvic organ prolapse.

### Statistical methods

Statistical analysis was performed using IBM SPSS Statistics for Windows, version 21.0. Count data were expressed as percentages. Categorical and continuous variables were subjected to χ^2^ and Mann–Whitney rank-sum tests, respectively. *P* < 0.05 was considered statistically significant.

## Results

### Injury to adjacent tissues

The incidence of injury to adjacent tissues was 7.68% (171/2226). Ureteral injury was the most common, followed by bladder, bowel, and vascular injuries, respectively (Table 2).

**Table 2:** Intraoperative injury to adjacent tissues (*n* = 2226)

Parameter	Number of patients, n (%)	
	Open surgery(*n* = 1254)	Laparoscopic surgery(*n* = 972)
Bladder injury	46 (4)	16(2)
Ureteral injury	51 (4)	17(2)
Bowel injury	28 (2)	10(1)
Vascular injury	3(0)	0(0)
Injury to adjacent tissues	128(10)	43(5)
Total incidence rate	171(7.68)	

### Short-term complications

The most common short-term complication was urinary tract infection (152/2226, 6.83%), followed by lymphocytes (139/2226, 6.24%), urinary retention (115/2226, 5.17%), perineal and lower-extremity oedema (93/2226, 4.18%), and surgical wound infection (61/2226, 2.74%). Occasional cases of intestinal obstruction, urinary incontinence, lymphatic leakage, deep vein thrombosis, ureteral fistula, and vaginal cuff dehiscence were also observed. The incidence of short-term complications was 31.45% (700/2226) (Table 3).

**Table 3:** Short-term complications (*n* = 2226)

Parameter	Number of patients, n (%)	
	Open surgery(*n* = 1254)	Laparoscopic surgery(*n* = 972)
Intestinal obstruction	29 (2)	10(1)
Perineal and lower-extremity oedema	68 (5)	25(3)
Urinary incontinence	31 (2)	12(1)
Urinary retention	85(7)	30(3)
Urinary tract infection	113(9)	39(4)
Lymphatic leakage	10(1)	4(0)
Lymphocyst	102(8)	37(4)
Surgical wound infection	47(4)	14(1)
Deep vein thrombosis	12(1)	4(0)
Ureteral fistula	19(2)	7(1)
Vaginal cuff dehiscence	1(0)	1(0)
Incidence of short-term complications	517(41)	183(18)
Total incidence rate	700(31)	

### Analysis of pelvic lymph node metastasis

The incidence of pelvic lymph node metastasis was 10.78% (240/2226). More specifically, none of the patients with stage IA2 cancer had lymph node metastasis, whereas the incidence for stage IB1, IB2, IIA1, and IIA2 were 12.74%, 15.69%, 9.42%, and 27.03%, respectively (Table 4).

**Table 4:** Pelvic lymph node metastasis/case (%) (*n* = 2226)

FIGO stage	No. of cases observed	No. of metastatic cases
IA2	310	0(0)
IB1	1499	191(13)
IB2	51	8(16)
IIA1	329	31(9)
IIA2	37	10(27)
Total incidence rate	240(11)	

### Postoperative pathological risk factors

In this study, 21.65% of patients had a tumour invasion depth ≥ 2/3, 3.37% showed parametrial invasion, and 13.39% were positive for LVSI (Table 5).

**Table 5:** Postoperative pathological risk factors, n (%) (*n* = 2226)

Parameter	Number of patients, n (%)
Pelvic lymph node metastasis	
N0	1986(89)
N1	240(11)
Resection margin of tumor	
R0	2221(99)
R1	11(1)
Parametrial invasion	
Positive	75(3)
Negative	2151(97)
Lymph vascular space invasion	
Positive	298(13)
Negative	1928(87)
Tumour size (cm)	
> 4	88(4)
≤ 4	2138(96)
Invasion depth	
≥ 2/3	482(22)
< 2/3	1744(78)

### Long-term complications

The most common long-term complication was urinary dysfunction, followed by bowel dysfunction and pelvic organ prolapse. The incidence of long-term complications was 2.96% (66/2226) (Table 6).

**Table 6:** Long-term complications, n (%) (*n* = 2226)

Parameter	Number of patients, n (%)	
	Open surgery(*n* = 1254)	Laparoscopic surgery(*n* = 972)
Urinary dysfunction	25 (2)	9(1)
Bowel dysfunction	21(2)	7 (1)
Pelvic organ prolapse	3(0)	1 (0)
Incidence of long-term complications	49(4)	17 (2)
Total incidence rate	66(3)	

### Analysis of univariate with the complications of RH + PLND

Analysis of short-term, and long-term complications of 937 cases with injury to the adjacent tissues showed that age, tumour size, invasion depth, parametrial invasion, LVSI, lymph node metastasis, FIGO stage, and surgical procedure were closely associated with the postoperative complications of RH + PLND (*P* < 0.05) (Table 7).

**Table 7:** Result of univariate analyses with the complications of RH + PLND, n (%)

Parameter	With complications	Without complications	χ2	*p*
Age, years				
20–40	103 (35)	195 (65)	50.4293	0.0000
41–50	315 (44)	395 (56)		
50–60	325 (51)	307 (49)		
61–72	194 (33)	392 (67)		
Other concomitant diseases				
Yes	276 (43)	361 (57)	0.5578	0.4551
No	661 (42)	928 (58)		
Pathological type				
Squamous cell carcinoma	876 (42)	1203 (58)	0.4954	0.7806
Adenocarcinoma	43(40)	65 (60)		
Adenosquamous carcinoma	18 (46)	21 (54)		
Pathological grade				
G1	51(38)	84 (62)	3.2663	0.3524
G2	570(41)	812 (59)		
G3	298 (44)	372 (56)		
Gx	18 (46)	21 (54)		
Tumour size (cm)				
≥ 4	81 (92)	7(8)	93.7495	0.0000
< 4	856 (40)	1282(60)		
Invasion depth				
≥ 2/3	236 (49)	246 (51)	11.9043	0.0006
< 2/3	701 (40)	1043 (60)		
Parametrial invasion				
Positive	41 (55)	34 (45)	5.0316	0.0249
Negative	896 (42)	1255 (58)		
Lymph vascular space invasion				
Positive	155 (52)	143 (48)	13.8841	0.0002
Negative	782 (41)	1146 (59)		
Pelvic lymph node metastasis				
With metastasis	126 (52)	114 (48)	11.9463	0.0005
Without metastasis	811 (41)	1175(59)		
FIGO stage				
IA2	80(26)	230(74)	162.6330	0.0000
IB1	590(39)	909(61)		
IB2	48(94)	3(6)		
IIA1	184(56)	145(44)		
IIA2	35(95)	2(5)		
Surgical procedure				
Open surgery	694 (55)	560 (45)	206.7357	0.0000
Laparoscopic surgery	243 (25.00)	729 (75.00)		

### Analysis of multivariate with the complications of RH + PLND

The age(61–72 years), invasion depth(< 2/3), LVSI negative and FIGO stage(IA2、IB1) could reduce the incidence of the postoperative complications of RH + PLND (Table 8).

**Table 8:** Result of multivariate analysis with the complications of RH + PLND, n (%)

Parameter	With complications	Without complications	OR(95% CI)	*p*
Age, years				
61–72	194 (33)	392 (67)	0.3(0.10–0.90)	0.030
Tumour size (cm)				
≥ 4	81 (92)	7(8)	4.58(1.40–15.01)	0.000
Invasion depth				
< 2/3	701 (40)	1043 (60)	0.15(0.04–0.52)	0.038
Parametrial invasion				
Negative	896 (42)	1255 (58)	0.18(0.05–0.62)	0.010
Lymph vascular space invasion				
Negative	782 (41)	1146 (59)	0.39(0.14–1.12)	0.080
FIGO stage				
IA2	80(26)	230(74)	0.49(0.36–0.67)	0.000
IB1	590(39)	909(61)	0.75(0.65–0.88)	0.000
IB2	48(94)	3(6)	4.67(2.01–10.83)	0.001
IIA2	35(95)	2(5)	2.49(2.12–8.96)	0.001
Surgical procedure				
Laparoscopic surgery	243 (25.00)	729 (75.00)	0.53(0.34–0.84)	0.008

### Classification of severity of complications

According to the Clavin-Dindo classification, there were no grade V patient deaths or grade IV life-threatening events in this study; grade IIIb adverse events were mainly intraoperative adjacent tissue injury and postoperative pelvic floor organ prolapse, and grade IIIa adverse events were mainly ureteral fistulas requiring endoscopic treatment and partial lymphatic cysts requiring treatment (Table 9).

**Table 9:** Classification of severity of complications for Clavin-Dindo (*n* = 937)

Parameter	Number of patients, n (%)
I	434(46)
II	302(32)
IIIa	29(3)
IIIb	172(18)
IVa	0(0.00)
IVb	0(0.00)
V	0(0.00)

## Discussion

Radical surgical procedure for cervical cancer involving RH (including the radical resection of the parametrium) and PLND (including the routine radical resection of pelvic lymph nodes), also known as Wertheim–Meigs operation. Which was regarded as the primary treatment method for cervical cancer [24]. In radical surgery for cervical cancer, the autonomic nerves of the ureters, bladder, and pelvic organs are prone to injury during the resection of pelvic organs, which can lead to bladder and rectal dysfunction [25, 26]. The incidence of complications associated with radical surgery for cervical cancer can be reduced by modifying the surgical approach, surgical procedure, and preservation of pelvic autonomic nerves [23, 27]. However, ureteral injury, bladder injury, surgical wound dehiscence, surgical site infection, urinary tract infection, and pelvic lymphocytes have occasionally been reported, as well as the frequent occurrence of complications related to the pelvic autonomic nervous system such as intestinal obstruction, urinary retention, urinary fistula, urinary incontinence, and sexual dysfunction [28, 29]. Some patients may require re-operation, which will prolong their hospital stay, increase treatment costs, and even cause permanent neurological sequelae or fatal consequences, thus severely affecting their quality of life.

With the introduction of laparoscopic surgery or laparoscopic robotic surgery to provide more options for cervical cancer surgical access and approach, its safety and efficacy are controversial [30, 31]. In Obermair’s research of early-stage cervical cancer, minimally invasive surgery was found to be associated with lower disease-free survival (4.5 years 86.0% vs. 96.5%, CI, -16.4 to -4.7) and overall survival (3-year rate, 93.8% vs. 99.0%, 95% CI, 1.77 to 20.30) [32]. Ramirez also reaffirmed that minimally invasive radical hysterectomy has a higher recurrence rate and a lower disease-free survival rate than open surgery [12]. In terms of postoperative adverse events, there was no significant difference between minimally invasive surgery and open surgery [32, 33]. In our study, there was a significant difference in the incidence of postoperative complications between minimally invasive surgery and open surgery, and similar to some previous studies, minimally invasive surgery reduced the incidence of postoperative complications, such as transfusion, wound infection, pelvic infection and abscess, lymphedema, intestinal obstruction, pulmonary embolism, deep vein thrombosis, and urinary tract infection [34, 35]. Laparoscopic radical hysterectomy (LRH) increase the incidence of ureteral injury and uterovaginal fistula in stage IB1 cervical cancer (FIGO 2009) with a tumour size less than 2 cm [36].

The incidence of postoperative adverse events in radical cervical cancer surgery was about 10.1–25.4%, while in the LACC trial the incidence was as high as 42%, while in our retrospective study the incidence of complications was 42.10% [12, 32]. The results of our study revealed that ureteral, bladder, and bowel injuries were common injuries to adjacent tissues. And the common short-term complications included urinary tract infection, lymphocyst, urinary retention, perineal and lower-extremity edema, and surgical wound infection, while the common distant-term complications included urinary dysfunction, bowel dysfunction, and pelvic organ prolapse. These common postoperative adverse events were similar in general to those described in previous studies [37, 38]. The result of this study showed high incidence rates of injury to adjacent tissues and short-term complications, most of which occurred before 2015. This may be related to the high proportion of cases undergoing open surgery and the proficiency of the newly established team of clinicians. In patients with other concomitant diseases, performing surgery after administering the proper treatment for these diseases did not increase the incidence of postoperative complications. In addition, tumor pathological type, degree of tumor differentiation were not necessarily related to the occurrence of complications. Age, tumor size, invasion depth, parametrial invasion, LVSI, lymph node metastasis, FIGO stage, and surgical procedure were risk factors for postoperative complications of RH + PLND.

A large proportion of the patients in this study were aged 40–60 years. This group of patients tended to have a later diagnosis, a higher degree of tumor malignancy, and greater surgical difficulty, which led to a higher rate of complications compared to the other two age groups. Tumors ≥ 4 cm, invasion depth ≥ 2/3, the presence of parametrial invasion, and the presence of LVSI led to a high rate of lymph node metastasis and advanced FIGO stage, which, in turn, elevated the surgical difficulty and, hence, the incidence of complications. Previous studies have reported that laparoscopic surgery can reduce the incidence of complications [32, 39]. Thus, surgeons who are highly proficient with open surgery can perform laparoscopic surgery to capitalize on the advantages of the latter, such as a smaller surgical wound, less bleeding, clear visual field, flexible operation, and quick postoperative recovery, thereby reducing the incidence of complications [40, 41]. Robot-assisted laparoscopy offers the advantages of a high-definition three-dimensional field of view, multi-dimensional flexible Endowrist movements, and stable tremor-filtered operations over simple laparoscopic surgery, which can better reduce the incidence of complications, shorten postoperative recovery time, and improve patient quality of life [42, 43].

The limitations of this study included the lack of a control group and randomisation, the lack of comparison between the inability to carry out adequate comparisons to RH + PLND performed at other centres. In addition, the differences in surgeon proficiency and the large time span of the study may also be confounding factors. The presence of numerous confounding factors also intervention logistic regression analysis of the factors associated with surgical complications, which also contributed to the limitations of this study. Furthermore, postoperative sexual dysfunction and psychological state were not assessed; thus, we could not evaluate patient quality of life at different time points after the surgery, nor the clinical characteristics affecting their quality of life. Thus, further analysis of postoperative quality of life could not be performed, which is another limitation of this retrospective study. Nevertheless, we believe that these data on complications add to our knowledge of the surgical complications associated with RH + PLND and further enrich the evidence and experience for preventing complications. Collating, summarizing, and learning from this evidence and experiences, and applying them to surgical procedures may reduce the incidence of complications in RH + PLND for cervical cancer and improve patient quality of life. Thus, with this rationale in mind, we hope that more future studies will address this topic.

## Conclusions

The incidence of cervical cancer remains high. The primary treatment method is multimodality therapy, of which RH + PLND is a key component. RH + PLND for cervical cancer is safe and practical. In this study, the most common injury to adjacent tissues was ureteral injury, the most common short-term complication was urinary tract infection, and the most common long-term complication was urinary dysfunction. Patients aged 40–60 years, with tumours ≥ 4 cm, invasion depth ≥ 2/3, parametrial invasion, LVSI, lymph node metastasis, FIGO stage > IB2, and who underwent open surgery were more prone to complications.

To reduce the incidence of complications, every effort should be made to foster proficient and excellent multidisciplinary diagnosis and treatment teams equipped with advanced technology; carry out cervical cancer screening to ensure the early detection, diagnosis, and treatment of cervical cancer; strictly control concomitant diseases during treatment; perform minimally invasive surgery where possible; conduct careful dissection and meticulous operations during surgery to spare the pelvic autonomic nerves and preserve the integrity of nerve conduction; and closely monitor patients after surgery. For patients with complications, individualized and careful treatment should be performed according to the characteristics of the complications, to improve their quality of life.

## Data Availability

Datasets are available on request from the corresponding author on reasonable request. The raw data and all related documents supporting the conclusions of this manuscript will be made available by the authors, without undue reservation, to any qualified researcher.
